# ‘Satan is holding your tongue back’: Stuttering as moral failure

**DOI:** 10.4102/ajod.v10i0.773

**Published:** 2021-04-23

**Authors:** Dane H. Isaacs

**Affiliations:** 1 Department of Psychology, Faculty of Arts and Social Sciences, Stellenbosch University, Stellenbosch, South Africa; 2 Human and Social Capabilities Division, Human Sciences Research Council, Cape Town, South Africa

**Keywords:** autoethnography, disability, discrimination, oppression, liminal nature, moral failure, South Africa, stuttering

## Abstract

**Background:**

The last decade has seen researchers and speech–language pathologists employ and advocate for a disability studies approach in the study of the lived experiences of people who stutter and in the design of interventions and treatment approaches for such individuals. Joshua St. Pierre, one of the few theorists to explore stuttering as a disability, mentions as a key issue the liminal nature of people who stutter when describing their disabling experiences.

**Objectives:**

This article aimed to build on the work of St. Pierre, exploring the liminal nature of people who stutter.

**Method:**

Drawing on my personal experiences of stuttering as a coloured South African man, I illuminated the liminal nature of stuttering.

**Results:**

This analytic autoethnography demonstrates how the interpretation of stuttering as the outcome of moral failure leads to the discrimination and oppression of people who stutter by able-bodied individuals as well as individuals who stutter.

**Conclusion:**

As long as stuttering is interpreted as the outcome of moral failure, the stigma and oppression, as well as the disablism experience by people who stutter, will continue to be concealed and left unaddressed.

## Introduction

Researchers and speech–language pathologists in the past decade have been employing and advocating for a disability studies approach in the study of the lived experiences of people who stutter and in the design of interventions and treatment approaches for such individuals (Boyle et al. 2016; Campbell, Constantino & Simpson 2019; Meredith 2019; Meredith & Packman 2015; Meredith, Packman & Marks 2012; St. Pierre 2018; Watermeyer & Kathard 2016; Wylie et al. 2013). St. Pierre (2012), one of the few theorists to explore stuttering as a disability, mentions as a key issue the liminal nature when describing the disabling experiences of people who stutter. With regards to the liminal nature of stuttering, St. Pierre (2012) argued that stuttering is not a homogenous phenomenon, but the fluency level of people who stutter fluctuates across different social contexts. For example, in certain social situations people who stutter may project almost fluent speech, whilst in other situations they may show significant levels of dysfluency (St. Pierre 2012). As a result, people who stutter possess what Watermeyer and Kathard (2016) termed as a spilt, complex disabled identity in society whereby they are neither clearly abled nor disabled. Therefore, it is commonly assumed that stuttering is not absolute and can be voluntarily controlled. Accordingly, expectations are often placed upon people who stutter to perform or communicate on the same level as able-bodied individuals (St. Pierre 2012).

These experiences frequently make people who stutter feel like ‘misfits’ (Garland-Thomson 2011). Garland-Thomson (2011) described misfitting as an incongruent relationship between the disabled individual and the expectations of the social environment. She argues that this incongruent relationship instantiates injustice and discrimination against disabled individuals (Garland-Thomson 2011). As a result of being ‘misfits’, people who stutter are typically unable to live up to the expectations of their social environment and face shame, embarrassment and oppression. For people who stutter, this oppression may also take the form of internalised oppression (Bailey, Simpson & Harris 2015). Bailey et al. (2015) argued that people who stutter commonly internalise the negative attitudes of stuttering that exists in society. As a consequence, individuals who stutter often harbour the most negative and harsh attitudes towards their dysfluent speech (Bailey et al. 2015). Disfluency ‘can thus be interpreted as a distinctly moral failure: the failure of a stutterer’s will and self-discipline which undercuts and threatens capitalistic virtues’ (St. Pierre 2012:3).

This research article builds on the work of St. Pierre (2012). Employing a methodology of analytic autoethnography, I reflect on different facets of my personal experiences as a person who stutters in order to shed light on the liminal nature of stuttering. I also illustrate how the interpretation of stuttering as the outcome of moral failure often leads to the discrimination and oppression of people who stutter by able-bodied individuals, as well as individuals who stutter. Disability scholars, such as Kittay (2019), Lourens (2018), Richards (2008) and Swartz (2014), have emphasised the value of the insider position within disability studies. These scholars argue that the insider position can provide critical insights into the lived and cultural experiences of disabled individuals – specifically into those physical and social structures, ideas, norms and cultural practices that oppress such individuals (Kittay 2019; Lourens 2018; Richards 2008; Swartz 2014).

## Autoethnography as a research method and design

Autoethnography can be defined as a form of research, writing or storytelling, which uses personal experiences to reveal the cultural, political and social aspects of phenomenon (Adams & Jones 2011; Ellis & Bochner 2000; Fa‘avae 2018; Maseti 2018). According to Ngunjiri, Hernandez and Chang (2010), autoethnography has three distinct features: firstly, autoethnography is a qualitative research method that approaches data collection, analysis and interpretation of self and social phenomenon regarding the self in a systematic manner. Secondly, autoethnography is self-focused: the researcher’s individual experience is the focus of the analysis. Finally, autoethnography is context conscious. Whilst there is strong focus on the self, autoethnography seeks to connect the self with the social context. More specifically, autoethnography seeks to understand the social context of the self and how the social context influences the construction of the self (Ngunjiri et al. 2010).

Autoethnography can take on many different forms (Ellis & Bochner 2000; Ngunjiri et al. 2010). Anderson (2006) made a distinction between two forms of autoethnography, namely, *evocative* and *analytic.* Evocative autoethnography involves the description of individual, emotional experiences. This form of autoethnography places great emphasis on the narrative and expressive skills which are demonstrated through art, such as poetry, prose and performances (Anderson 2006). Analytic autoethnography describes the researcher’s subjective experiences, with the aim of formulating theoretical understanding of wider social phenomena (Anderson 2006). The autoethnography presented in this article is an analytic autoethnography, and I use retrospective recollection to illuminate key theoretical issues. I have suggested elsewhere that ‘a retrospective recollection is an explicitly subjective and qualitative approach that utilities the researcher’s personal memories and lived experience as material for analysis’ (Isaacs 2020:60). In the current article, I recollect and examine my personal experiences of stuttering as a coloured South African man, across different stages of my life, through a disability studies lens. Through this analysis, I seek to illuminate the liminal nature of stuttering, which results in an embodied experience of moral failure. As highlighted earlier is this article, the liminal nature of stuttering and its interpretation as moral failure are essential for understanding the disabling nature of stuttering (St. Pierre 2012).

## Satan, studying and shopping for cures: My personal journey

It is suggested that stuttering does not start at birth, but that individuals begin to stutter during early childhood between 2 and 6 years of age (Ezrati-Vinacour, Platzky & Yair 2001; Vanryckeghem, Brutten & Hernandez 2005; Woolston 2019). My first conscious memory of stuttering was in Sub A (now known as Grade 1 in the South African schooling system, the first year of formal academic schooling). I was requested by my teacher to read aloud and I could not. I stuttered on a few words, but eventually I managed to read the text. I did not understand what was happening at the time. When I asked my mother why this had happened, she told me one of infamous myths commonly associated with stuttering (National Stuttering Association [NSA] 2020). She replied that I had imitated another child at crèche, and that was the reason why I started stuttering (NSA 2020). She eventually took me to a speech therapist. The therapist told my mother that I had a stutter, and that I should breathe slowly before I speak. Because I grew up in an evangelical Christian home, I was also taken up for prayer continuously to receive healing, but there was no improvement. I was told that I did not have enough faith to be healed. I was also told that although God wanted to heal me, He could not because Satan was holding my tongue back. I needed to seek God and ask Him what in my life allowed Satan to gain control over my speech. Even to this day, when I come into contact with evangelical Christians, there is always the need (on their part) to pray for me or rebuke my stutter. This experience is not unique to my story, but is all too familiar for many disabled individuals, particularly those who form part of evangelical Christian communities. For example, in recent studies conducted by Sande (2019) and Stanley (2019), they found that impairments are typically viewed as a test of faith that can be overcome through divine healing. Therefore, disabled individuals are frequently encouraged to put their faith in action and receive the healing they desire. The disabled individual’s inability to receive healing is commonly interpreted as a consequence of unbelief, demonic influence or the presence of sin (Sande 2019; Stanley 2019). Nevertheless, as the time progressed, the liminal nature of my stuttering became more apparent. St. Pierre (2012) argued that because of the liminality of stuttering, individuals are not clearly identified as disabled or able-bodied. As a result, they are commonly expected to perform on the same level as abled-bodied individuals (St. Pierre 2012).

Similar to many individuals who stutter, the liminal nature of stuttering caused my family to see my stutter as an invisible problem (Butler 2013a; Scharf 2017). I was continually told, ‘[*t*]here is nothing wrong with you’; ‘[*s*]peak slowly’; and ‘[*t*]ake a deep breath before you speak’. I was expected to perform at the same level of my two siblings who are fluent speakers. I was required to answer the house telephone and was expected to go to the neighbourhood shop despite spending most of the journey to the shop anxiously practising what I needed to say in order to avoid stuttering in front of the shop attendant. But for much of this stage of my life, my stutter was controllable and I could conceal it. Throughout my primary school career, I could fulfil the role of an able-bodied person as my stutter was not severe. I managed to do what were termed ‘orals’, I read aloud in class without much difficulty, and I was quite the extrovert.

When I started high school, the severity of my stutter started showing its ugly head. Previous research has emphasised the harsh bullying children who stutter commonly endure during their schooling career (Butler 2013a; Davis, Howell & Cooke 2002; Hughes 2014; Kikuchi et al. 2019). As a result of bullying, children who stutter typically become withdrawn, feel isolated, have a reduced self-esteem and have poor peer relationships (Butler 2013a; Davis et al. 2002; Hughes 2014; Kikuchi et al. 2019). For me, the first semester term of high school went well, but in the second semester my stutter became severe because I became a victim of bullying. This bullying exacerbated my stutter to the extent that by the second year of high school (Grade 9), I became completely withdrawn. I spent most of my intermission periods alone. I had a very low self-esteem. I was forced to tell my teachers about my stutter and request that I do my orals and reading after class. Many of the teachers were accommodating, whilst others, concerned with my future success, encouraged me to go to a speech therapist again to get my stutter ‘under control’. I finally decided to go to a speech therapist in Grade 11.

Whilst the last decade has seen speech–language pathologists develop interventions to address the psychosocial needs of people who stutter, scholars, such as Watermeyer and Kathard (2016) argue that at the centre of several intervention strategies, is the reduction of dysfluency and the promotion of fluency. Watermeyer and Kathard (2016) explain because of the intense experiences of oppression and discrimination, clients who stutter have a strong desire ‘to get better’ and end the cycle of oppression and discrimination. It is at times difficult for clinicians to remain mindful of the negative implication of this position. In response, they design intervention strategies to reduce dysfluency and promote fluency (Watermeyer & Kathard 2016). This was my experience of speech therapy. As my ultimate aim for attending speech therapy was to gain control over my stutter, the speech therapist taught me various breathing techniques, which I still use to try to exercise control over my stutter. I was encouraged to apply these techniques to any and every oral situation I was faced with. The application of these techniques were closely monitored by the speech therapist. After each session, my level of fluency was measured according to a fluency scale. If my fluency was not up to the standard, the speech therapist would at times scold me and encourage me to do better next time. Once I mustered up enough confidence, I braved the fear of stuttering, and attempted an English oral, where I failed horribly and was deeply embarrassed. My teacher was supportive and commended my bravery, but some of my classmates felt that I had wasted their time. My life seemed to be a series of blocks – one after another. I became wary about doing orals in the future. Eventually, I had to stop speech therapy because my academic commitments became too demanding. For the remainder of my schooling career, I was permitted to avoid doing all oral activities.

During my undergraduate studies at university, I was also able to escape class presentations. The large size of the classes provided me with possibilities to skip classes where oral assessments would be carried out. So, I was able to keep up the performance as an able-bodied individual. This experience coincided with a study conducted by Butler (2013a) on the progression of people who stutter into higher education. Butler (2013a) found that participants enjoyed university in comparison with school. The large cohort of students removed the pressure for asking questions, to do presentations and participate in seminar discussions. However, my first year of postgraduate studies (my Honours year) was the worst year of my academic life (see Isaacs 2020). Oral assessments were at the core of the course. Owing to my stuttering being sporadic and not recognised as a disability, I was not allowed to be disabled. I felt that I was constantly moving between the identity of an abled man and that of a disabled man. Whilst I navigated between both identities, the structure of the course required that I fulfil the role of an abled-bodied (fluent) individual. We were graded for class participation and were expected to run seminars. It was really difficult, but I had to try my utmost to be fluent. The more I tried to be fluent, the more severe my stutter became. Many times, I felt like an invalid and experienced many depressive episodes. I attended counselling for these depressive episodes and sought Christian counselling, but nothing seemed to help. At the end of my Honours year, I had been rejected for both the Master’s degrees in both clinical and research psychology, and I was not able to get a placement to do my counselling internship (see Isaacs 2020).

Fortunately, I was accepted to carry out a research masters by thesis only at another university. Before accepting the offer, I met my supervisor and explained my negative experiences of stuttering at the previous university. I asked my supervisor if my stutter would be a problem. She assured me that it would not be an issue. She tried to make the course as accommodating as possible. For instance, instead of me performing a conventional oral presentation of my Masters’ research proposal in front of the admissions committee, she consulted the university’s disability unit about alternative techniques we could use to present my research. We decided that it would be best if I do not perform an oral presentation, but instead submitted my proposal electronically in written form to the committee and request that they email their questions to me, and I would respond in writing accordingly. Also, because of my stutter being so severe at the time, she gave me the option of either performing a media analysis for my data collection or sending me for interview training if I wanted to use interviews as a method of data collection for my research. We decided that a media analysis would be a more suited option. A staff member in the department expressed reservations about me performing a media analysis, stating that conducting interviews is a much more valuable skill at Master’s level. She attributed my stutter to anxiety. She said that she herself had struggled with anxiety during high school but had overcome this. She believed that it was important that I overcome my stutter, because fluency was an important requirement for success in academia.

The 2 years I spent doing Masters were fairly relaxed. There was no pressure to perform any oral assessment. Shortly after I submitted my Master’s thesis for examination, I applied for an internship at a science council in Cape Town. My application was successful. Although I was excited about the internship, on entering the science council I was aware that fluency was central to achieving success and promotion in such a space. I had very supportive colleagues who tried to make the space as comfortable and accommodating for me as possible. However, the culture of the organisation did not allow that I position myself as a disabled man. As I was not physically impaired and my stutter allowed me to pass as a fluent speaker in certain situations, I was expected to carry out the same oral activities as the fluent interns. At times, colleagues would jokingly say, ‘[*t*]here’s nothing wrong with you – it’s all in your head. You can socialise and make jokes without stuttering. So, pull yourself together’. From the liminal nature of my stutter, listeners felt that I could attain fluency if I worked hard enough at it. Some colleagues suggested that I attend speech therapy; others would share stories of people who put in the necessary hard work and overcame their stutter. These stories shared motivated me to work and eventually overcome my stutter. Therefore, for the duration of my time spent at the science council, I worked hard to pass as fluent. At times, I succeeded, but many times I failed horribly. Each time I failed, I would blame myself for not working hard enough. Many colleagues would commend my bravery. Some interpreted the stuttering as me still being stuck in what they termed as ‘victim mentality’. On one occasion, a colleague even questioned my suitability for the organisation, and suggested that I had chosen an incorrect career path.

Interestingly, this idea of stuttering as the outcome of moral failure has been held not only by fluent speakers but also reproduced by people who stutter. As stated previously, Bailey et al. (2015) and colleagues noted that stuttering commonly attracts harsh and negative societal responses. These responses are typically absorbed by people who stutter. As a result, people who stutter may be particularly negative in their response to dysfluent speech (Bailey et al. 2015). Therefore, there typically exists a strong desire to gain control over stuttering, renounce the stigmatised identity of stuttering (Butler 2013b) and, in turn, attain the identity of an abled-bodied, fluent individual (Watermeyer & Kathard 2016). Several people I have met who have managed to gain control over their stutter through speech therapy and/or self-help groups have dissociated themselves from the disabling identity and disabling nature of stuttering. They have aligned themselves with cultural norms and ideas of fluency, arguing that exercising controlled speech is the only way to gain true acceptance in society. Yes, they seem to say, you are encouraged to disclose that you are a person who stutters, and at times request extra time when you are expected to deliver a presentation in a professional setting; however, under no circumstances can society accept disfluency.

Similar sentiments were shared at a for-profit self-help course I attended for people who stutter. I was introduced into this course by men who stutter and who participated in my doctoral study. I received only good testimonials from the men who participated in the course. Yet, I was sceptical about attending the course because of previous speech therapy sessions that did not yield the desired result of overcoming my stutter and becoming a fluent speaker, but I decided nevertheless to give it a try. Attending the course was a good experience. As it was run by people who stutter, the course was designed to address the psychosocial needs of people who stutter. On the course we were assured that stuttering was not a disability. Instead, we were introduced to specific techniques to help us gain control over our stutter, particularly during orally challenging situations. Similar to other new students, I left the course feeling cured and in control of my stutter. As a way to ensure that we maintain the correct usage of speech, we were required to attend weekly support groups. During these sessions, we would share our successes using the techniques we learnt on the course during challenging social situations. In the same way, there were stories where participants lost control over their stutter. Every time I heard these stories, I would be disappointed and witness how these men (the graduates predominately consist of men who stutter) would fight against this concealed weakness and vulnerability. However, they were determined to master the techniques learnt and combat the stigmatised identity of an individual who stutters (Butler 2013b).

## Discussion

As outlined above, over the course of my life, the liminal nature of my stutter has been viewed as a speech problem I could and should exercise control over. The language of moralising in terms of lack of control has changed from ‘the work of Satan’ to an appeal from teachers, lecturers, colleagues and a for-profit company that I exercise the kind of control over my life expected from what, in the contemporary neoliberal context, has been termed as the ‘responsibilized’ subject (Chaudhry 2018; Colvin, Robin & Leavens 2010; Trnka & Trundle 2014). There was an expectation that I reject the identity of a disabled person and perform on the same level as an able-bodied individual (St. Pierre 2012). These experiences frequently made me feel like a ‘misfit’ in relation to my social environment (Garland-Thomson 2011), with much of my not fitting in being described in implicitly moral terms. Whilst there were times, I could uphold the performance of an able-bodied individual; however, in many situations I would lose control over my stutter. This would be interpreted distinctly as moral failure, which led to recurring incidents of discrimination and oppression. As my stutter was viewed as the outcome of moral failure, there was a belief that with the correct self-help group and sufficient speech therapy, I could manage and gain control over my stutter. The issue of control and self-control, interesting enough, features in both the religious discourse and contemporary neoliberal social arrangements.

Furthermore, my personal experience of stuttering outlined how interventions for stuttering may sometimes also view and approach stuttering as moral failure, or a problem that can be fixed, controlled and managed (St. Pierre 2012). This one-dimensional focus places the responsibility of stuttering completely on the individual. It defocuses from the oppression people who stutter experience in attempting to perform and maintain eloquent and fluent speech (St. Pierre 2019). A consequence of adopting a moralistic approach in the design and implementation of interventions, is that people who stutter, may experience shame at not being able to exercise control over their stutter. The language of a popular stuttering intervention programme is instructive here. According to McGuire (2014):

[*Y*]ou [the person who stutters] will have certain sounds and words that trigger more fear than others, resulting in, you know FSD (freeze, struggle and distort). You must attack these (thereby the fear/panic) with the weapons you’ve just learned. Not only attack but extinguish, kill, wipe out, etc. until you’re bored with it … Bored means 100% confident – 0% fear. (p. 57)

The language used here is prescriptive and militaristic. The expectation to combat dysfluency and to strive towards fluent speech in the light of the liminal nature of stuttering may lead to internalised oppression (Watermeyer & Görgens 2014). Watermeyer and Görgens (2014) explained that cultural ideas and attitudes shape disabled people’s own subjectivity and self-perceptions. As disabled people fear being stereotyped as dependent, weak or helpless, they may grow into assuming an in control public persona in order to obtain affirmation, which drives them away from self-discovery and self-acceptance. The strong need for upholding the accepted public persona may have negative implications for the psychological well-being of the disabled individual, leading to self-doubt, identity confusion, feelings of inferiority and mental health problems (Watermeyer & Kathard 2016). These issues, as Watermeyer and Görgens (2014) suggested, may affect all people with disabilities. In the case of stuttering, the effects may be even more impactful. As long as stuttering continues to be interpreted as the outcome of moral failure, the complexities associated with the disabling experience of stuttering, the stigma and oppression attached to it, continue to be concealed and left unaddressed.

It is thus essential that the interpretation of stuttering should transform from viewing stuttering as a moral failure into understanding stuttering in its social and political context – in short, as using what is known theoretically about disability and disablism to understand the experience of disability. A transition towards a disability studies approach will help to unearth the social and disabling nature of stuttering (Campbell et al. 2019). Specifically, it is crucial to understand how individuals who stutter are disabled by their social environment – more importantly by those dominant ideas and practices of communication, and oppressive attitudes and stigmas of dysfluency (Bailey et al. 2015; Bricker-Katz, Lincoln & Cumming 2013; St. Pierre 2017). Over and above this, adopting a disability studies approach is important for transformation (Kafer 2013; St. Pierre 2019). As illustrated in my autoethnography, spaces of basic and higher education, as well as spaces of employment, demand verbal fluency. This demand for verbal fluency is exclusionary and discriminatory for people who stutter. Spaces such as those mentioned above need to approach and engage with stuttering through a disability studies lens. This would cultivate conversation and promote the social inclusion and the constitutional and human rights of individuals who stutter. However above this, it would challenge those ableist norms and ideas dominating these spaces, and allow for diversity and a place for disability (St. Pierre 2019).

In the same way, a disability studies approach is also likely to be beneficial in the design of intervention strategies for people who stutter. Indeed, there has been concerted efforts by professionals to address the disabling needs of people who stutter through environmental, functional and biopsychosocial models of disability (Boyle 2019). In a recent book, *Stammering Pride and Prejudice: Difference not Defect*, Boyle (2019) called for the collaborative work between professionals and disability rights advocates to further strengthen and design effective strategies in order to improve public attitudes and responses to people who stutter. According to Boyle (2019), professionals commonly approach stuttering from a service agenda framework, which includes using therapy to address self-stigma. Whilst therapy has been critical in addressing and reducing stigma amongst people who stutter, I, similar to Boyle (2019), recommend that professionals and researchers extend their scope of focus and take a more active role in reducing stigma through advocacy at a political and institutional level. For instance, these include advocating for the formulation of policies that promote the social inclusion of people who stutter, and modifying environmental barriers to accommodate diversity and the equal participation of such individuals (Boyle 2019). In addition, professionals and researchers could be instrumental in alerting and educating families, communities and the greater public about the prejudice, stigma and discrimination fashioned against people who stutter (Boyle 2019). In this way, we may be closer to effectively responding to and opposing the prejudice, stigma, discrimination and oppression commonly faced by people who stutter.
